# Bidirectional modulation of soleus spinal excitability induced by upper limb movement practice

**DOI:** 10.1007/s00221-026-07299-6

**Published:** 2026-04-27

**Authors:** Aditi Doshi, Ananya Handa, Trisha Kesar, Sangeetha Madhavan

**Affiliations:** 1 Brain Plasticity Laboratory, Department of Physical Therapy, College of Applied Health Sciences, University of Illinois Chicago, 1919 W. Taylor St., Chicago, IL 60612 USA; 2 Graduate Program in Neuroscience, University of Illinois Chicago, Chicago, IL USA; 3 Department of Rehabilitation Medicine, Division of Physical Therapy, Emory University, Atlanta, GA USA; 4 Department of Physical Therapy, College of Applied Health Sciences, University of Illinois Chicago, Chicago, IL USA

**Keywords:** Interlimb neural coupling, Soleus H-reflex, Spinal excitability, Upper limb movement, Neuromodulation

## Abstract

Interlimb neural coupling allows upper limb (UL) activity to influence lower limb (LL) spinal excitability, yet the direction and magnitude of this modulation can vary across individuals. While rhythmic, locomotor-like UL tasks typically suppress soleus H-reflex amplitude, the effects of discrete voluntary UL movements remain unclear. The objective of this study was to determine whether rhythmic UL movements modulate soleus H-reflex excitability. Twenty-four able-bodied adults completed a single session including a 20-min paradigm of four symmetric, bilateral UL movements performed at 1 Hz. Soleus H-reflexes were measured using peripheral nerve stimulation while participants stood upright (two baselines and post UL task). H_max_/M_max_ ratios were compared across time points, and k-means clustering was applied to individual change scores to identify responder subgroups. No significant group-level modulation of H-reflex excitability was observed following UL movement practice (*p* = 0.564). However, clustering revealed two distinct and opposite response profiles. One subgroup (n = 13) demonstrated a significant decrease in H-reflex excitability (22.1% ± 11.0% suppression), while the other subgroup (n = 11) exhibited a significant increase (19.5% ± 12.5% facilitation). Fatigue ratings increased post-intervention, whereas energy ratings remained stable. UL movements can modulate spinal excitability bidirectionally, with individuals showing either facilitation or suppression of the soleus H-reflex. This individualized responsiveness highlights heterogeneity in interlimb neural coupling, suggesting that UL-based neuromodulation strategies may require personalized tailoring. The classification of facilitatory and suppressive responders offers a methodological framework for developing adaptive rehabilitation approaches that leverage natural interlimb circuit interactions.

## Introduction

Coordinated human movement relies on seamless sensorimotor integration across the central and peripheral nervous systems (Zehr and Duysens 2004). This sensorimotor integration is particularly evident during rhythmic locomotor behaviors, such as walking and running, where both upper and lower limbs contribute to a coordinated output involved in static and dynamic balance, postural control, and the generation of propulsion during gait. A central mechanism underlying sensorimotor coordination is interlimb neural coupling, a process in which activity in one limb (e.g. upper limb) modulates motor neuron excitability in distant limbs (eg. lower limb) through neural pathways linking cervical and lumbosacral spinal segments (Arya and Pandian 2014; Frigon et al. 2004; Zehr et al. 2016). This coupling is clearly demonstrated during human gait, where rhythmic arm swing is phase-locked to contralateral leg movement and contributes to stability, propulsion, and the overall efficiency of walking. Perturbing or enhancing arm swing can alter lower-limb muscle activity and step timing, indicating that upper-limb movements exert a functional influence on locomotor output through shared cervical–lumbar neural pathways (Zehr 2005; Zehr and Haridas 2003; Zehr and Duysens 2004). These pathways, conserved from our quadrupedal ancestry, remain functionally significant in bipedal humans (Zehr 2005; Zehr et al. 2009). For example, limiting arm swing, such as when carrying objects, has been shown to increase step width and reduce gait efficiency, underscoring that these neural linkages shape everyday locomotor behavior (Collins et al. 2009). Given the functional relevance of these interlimb circuits, understanding how upper limb (UL) movement influences lower limb (LL) excitability is critical for advancing neurorehabilitation and motor retraining strategies.

The Hoffman’s (H-)reflex is a well-established non-invasive tool for probing spinal reflex circuit excitability, reflecting the responsiveness of the monosynaptic reflex arc between the Ia sensory afferents and lower motor neurons, and for evaluating reflex modulation by both peripheral and descending inputs (Palmieri et al. 2004). The soleus H-reflex offers a reliable and sensitive measure that is modulated by muscle activation, posture, and task demands (Burke 2016; Knikou 2007; Zehr 2002). Numerous studies have demonstrated that UL activity can alter soleus H-reflex amplitude, providing evidence of functional interlimb communication (Frigon et al. 2004; Javan and Zehr 2008; Palomino et al. 2011; Solopova et al. 2017; Zehr et al. 2004).

During rhythmic, locomotor-like UL tasks, such as arm cycling, soleus H-reflex amplitude is typically suppressed, likely due to increased presynaptic inhibition of Ia afferents (Chen and Zhou 2011; Frigon et al. 2004; Palomino et al. 2011; Zehr and Duysens 2004). Several studies have documented facilitatory, suppressive, or negligible effects on soleus H-reflex amplitude when upper limb movements deviate from rhythmic, locomotor-like patterns, particularly during asymmetric, low-frequency, or non-weight-bearing tasks involving the wrist or hand (Delwaide et al. 1977; Javan and Zehr 2008; Solopova et al. 2017; Toth et al. 2022). For example, Solopova et al. (2017) demonstrated that rhythmic wrist flexion increased soleus H-reflex amplitude under unloaded conditions, whereas adding resistance suppressed the reflex, highlighting the influence of task-specific sensory input. Similarly, Toth et al. (2022) showed that cutaneous stimulation of the hand modulates ankle muscle activity, emphasizing the role of afferent feedback in interlimb coupling.

However, variability in outcomes is not solely attributable to task differences. Across studies, participant characteristics such as age, sex, and neurological status vary considerably and are known to influence H-reflex excitability (Burke 2016; Knikou 2008). Importantly, inconsistent responses—including facilitation, suppression, and no change—have also been reported within similar experimental paradigms and in neurologically intact populations (Barzi and Zehr 2008; Zehr et al. 2016, 2004), suggesting that task parameters alone do not fully account for the observed variability. Instead, these findings point to individual-specific modulation patterns, potentially reflecting differences in spinal circuitry, descending control, or sensorimotor integration strategies.

Despite growing evidence, most prior studies have focused on cyclic, locomotor-like UL movements, with limited investigation of discrete, voluntary upper limb actions more representative of daily activities and clinically-relevant functional movements. Additionally, group-averaged analyses may mask meaningful interindividual variability in reflex modulation (Kato et al. 2022; Nakajima et al. 2013). Identifying distinct individual-specific interlimb coupling response patterns could provide novel insights into personalized neuroplasticity profiles relevant to rehabilitation.

The present study introduces a novel paradigm involving four symmetric, discrete bilateral UL movements paced at 1 Hz. Our design enables precise temporal control of UL while mimicking clinically relevant movement patterns. Using this model, we aimed to (1) determine whether discrete UL movements modulate soleus H-reflex amplitude in healthy adults, and (2) identify subgroups exhibiting distinct patterns of facilitation, suppression, or no change using clustering analysis. The long-term goal of this study is to enhance our understanding of functional interlimb coupling and inform individualized approaches to neuromodulation and motor recovery.

## Materials and methods

### Participants

Twenty-five able-bodied adults (13 female, 12 male; *M*ean = 25.9 years, *StDev* = 4.2, Range = 20–37) participated in the study (see Table 1). Inclusion criteria included being between 18 and 40 years of age and having no history of neurological disorders (e.g., epilepsy, multiple sclerosis, Parkinson’s disease, stroke, traumatic brain injury) or musculoskeletal injuries affecting study participation. Exclusion criteria included untreated cardiovascular, pulmonary, or metabolic conditions; active infections; sensory neuropathy; recent surgery on the non-dominant leg; or skin sensitivity such as allodynia or hyperalgesia. All participants provided written informed consent prior to participation. The study protocol was approved by the University of Illinois Institutional Review Board and conducted in accordance with the Declaration of Helsinki.

**Table 1:** Participant characteristics

*Characteristics*	
Total participants	25
Mean age (years)	25.9
Age range (years)	20–37
Sex (Males)	13
Handedness (Right, %)	88%
Leg tested (Left, %)	84%

### Experimental design

**Fig. 1:**
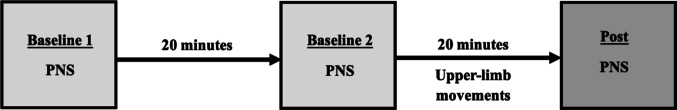
Schematic of the study design. Each participant underwent two baseline soleus H-reflex recordings separated by a 20-min interval. Soleus H-reflexes were elicited via peripheral nerve stimulation of the tibial nerve in the popliteal fossa while the participant was seated at rest. Following baseline measurements, participants completed the upper limb (UL) movement task, after which post-intervention soleus H-reflexes were recorded using the same stimulation parameters

Participants completed two baseline soleus peripheral nerve stimulation (PNS) measurements (Baseline 1 and Baseline 2) separated by a 20-min rest period, followed by a 20-min upper limb (UL) movement task, and a final post-intervention PNS measurement (Post) over the course of one session (see Fig. 1). This design allowed for the evaluation of baseline reliability and post-task modulation of spinal H-reflex excitability. The non-dominant leg (the leg not preferred for kicking a ball) was tested for all participants, with 84% of participants using the left leg. Perceived fatigue and energy were evaluated before and after the experimental protocol using the Visual Analog Scale for Fatigue (VAS-F) (Lee et al. 1991). Throughout all experimental procedures, participants remained seated with their legs in a stationary position.

### UL movement task paradigms

**Fig. 2:**
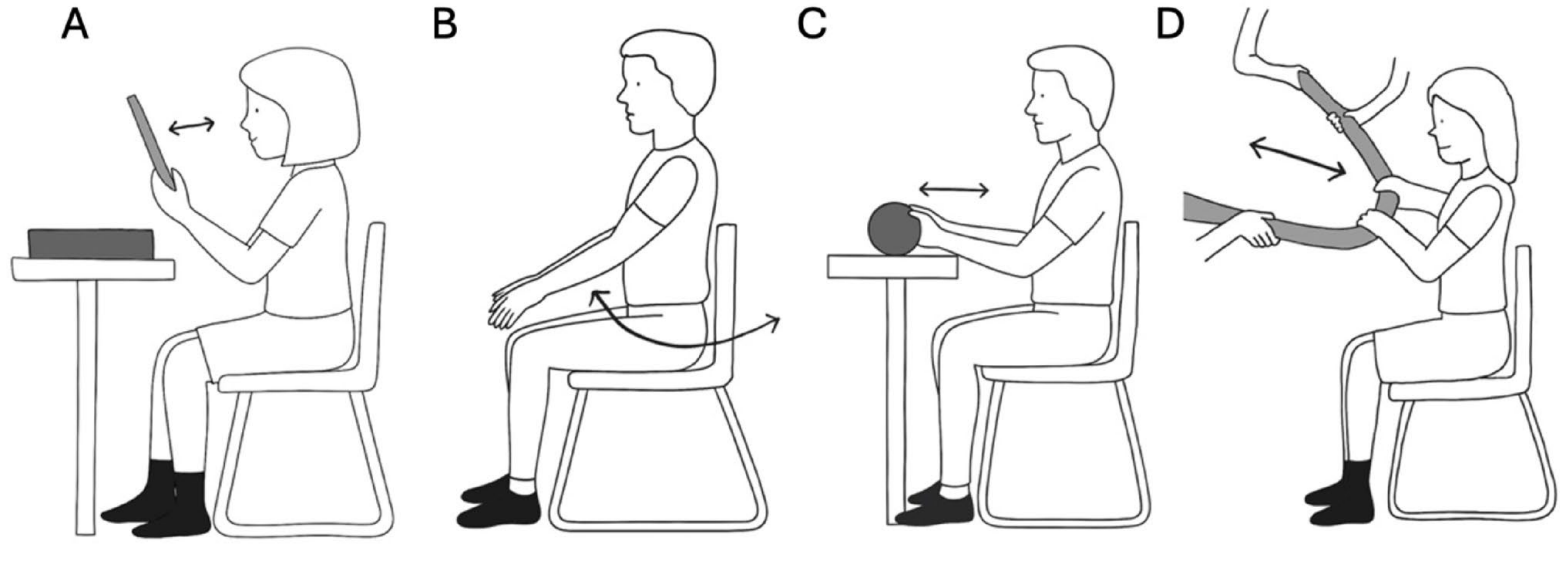
Upper limb (UL) movement paradigm. Participants performed four discrete, rhythmic, and symmetric bilateral arm movements paced at 1 Hz using a metronome. This paradigm was designed to mimic clinically relevant voluntary motor actions rather than cyclical locomotor patterns. Participants performed four different tasks: A: Playing Drums, B: Arm Swing, C: Rolling, and D: Rowing (The experimenters held the apparatus; no external resistance was applied)

Participants performed four discrete, symmetric, and rhythmic bilateral UL tasks paced at 1 Hz using a metronome (see Fig. 2):

- *Playing Drums*—rhythmic drumming movements using a wooden stick (based covered in gripping foam for easier grip) with coordinated wrist, elbow, and shoulder motion; and
- *Arm Swing*—alternating forward and backward arm swings to approximately 90° of shoulder flexion;
- *Rolling*—rolling a foam log (18 inches) back and forth, involving shoulder and elbow flexion/extension;
- *Rowing*—simulated rowing involving coordinated shoulder and elbow flexion/extension at a 90° shoulder angle.

Each task lasted 5 min, with 20 min total for the 4 tasks. The task order was randomized for each participant. Participants remained seated with their backs supported and their feet stationary and touching flat on the floor to ensure that movement was restricted to the UL. These tasks were designed to replicate voluntary, clinically relevant arm movements rather than cyclic locomotor patterns.

### Methodology for measuring soleus H-reflexes

Surface electromyography (EMG; Biometrics SX230, Newport, UK) was recorded from the soleus muscle of the non-dominant leg, with a ground electrode placed on the wrist. EMG signals were band-pass filtered (10–500 Hz), amplified (1000×), and sampled at 2,000 Hz. Electrical stimulation (Digitimer DS7A, Hertfordshire, UK) was delivered via a 5-cm round cathode at the popliteal fossa and a 5 × 5 cm anode on the quadriceps tendon. Single 1-ms PNS pulses were applied at random inter-pulse intervals (5–8 s). Participants stood upright during the procedure with equal weight distributed between both legs. The tested (non-dominant) leg was maintained in a neutral position with the knee slightly flexed and the ankle in a natural, relaxed posture. Participants were instructed to remain relaxed and avoid any voluntary contraction of the soleus muscle throughout testing. Background EMG activity was monitored to ensure minimal muscle activation prior to each stimulus.

PNS stimulation intensity was gradually increased to generate an H-reflex recruitment curve, during which multiple stimuli were delivered across a range of intensities to identify the maximal H-reflex amplitude (Hmax) and the maximal direct motor response (Mmax) amplitude. As the recruitment curve was constructed individually for each participant, the total number of stimuli varied depending on the resolution required to accurately determine peak responses. The H_max_/M_max_ ratio was calculated and served as the primary index of spinal excitability. PNS electrode placement and response morphology (M-wave latency: 6–9 ms; H-reflex latency: 30–40 ms) were verified before securing the electrodes. Baseline measurements were collected prior to each task to account for potential time-dependent fluctuations in H-reflex excitability and to provide a within-subject reference for quantifying task-related modulation. Additionally, to reduce variability, stimulation parameters and electrode placement were kept constant throughout testing, and responses were visually inspected to ensure consistent M-wave and H-reflex morphology across trials. Procedures followed established H-reflex recording methods (Burke 1999; Hoque et al. 2018; Hoque et al. 2019; Knikou 2008; Zehr and Stein 1999).

### Fatigue and energy rating

Subjective fatigue and energy were assessed using the VAS-F (Lee et al. 1991), consisting of 18 items rated on a 0–10 numeric scale (13 fatigue items, 5 energy items). Participants completed the VAS-F immediately before and after the experiment. Higher subscale totals indicated greater perceived fatigue or energy use, respectively.

### Data and statistical analyses

Data were processed and analyzed in R (R Core Team 2024) using the packages *dplyr*, *rstatix*, *cluster*, and *factoextra*. Peak-to-peak amplitudes of soleus H-reflex and M-wave responses were averaged across the three largest responses for each condition. The soleus H_max_/M_max_ ratios were calculated for Baseline 1, Baseline 2, and Post. The two baseline measures were averaged to create a single stable baseline value (AvgBaseline). The primary dependent variable was spinal excitability, quantified as the soleus H_max_/M_max_ ratio. Change scores for the H_max_/M_max_ ratio (Post − AvgBaseline) were computed to assess individual responses to the UL intervention. The VAS-F subscales for fatigue and energy were summed separately at pre- and post-assessment. All data were retained to preserve interindividual variability; no outlier removal was performed.

Data normality was evaluated using the Shapiro–Wilk test. Because most variables violated normality assumptions, nonparametric statistics were employed with an α level of 0.05. Baseline reliability was assessed using Wilcoxon signed-rank tests, comparing Baseline 1 and Baseline 2. To evaluate the overall intervention effect, Wilcoxon signed-rank tests were used to compare AvgBaseline and Post values. To explore potential responder subgroups, k-means clustering was performed on individual change scores. Cluster validity was examined using Mann–Whitney U tests comparing subgroup distributions. Within each identified cluster, additional Wilcoxon signed-rank tests compared AvgBaseline and Post values to assess within-group changes. Pre–post differences in VAS-F fatigue and energy scores were also evaluated using Wilcoxon signed-rank tests.

## Results

Of the twenty-five healthy participants enrolled, 24 completed all study procedures, including two baseline sessions, the UL movement task, and the post-intervention H-reflex assessment. One participant withdrew due to apprehension about the experimental procedures.

**Fig. 3:**
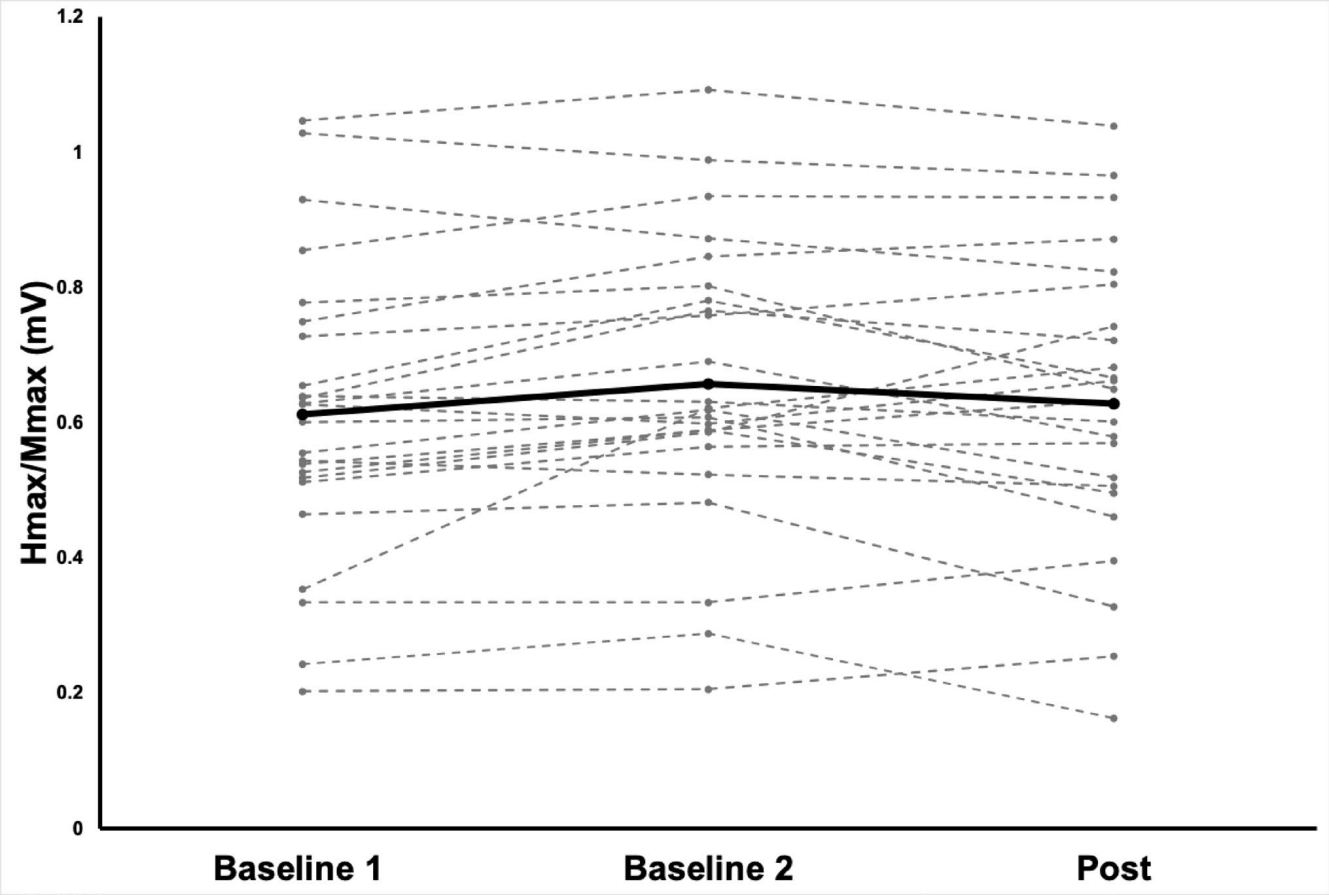
Individual H-reflex excitability across Baseline 1, Baseline 2, and Post conditions. H_max_/M_max_ values across two baseline assessments and post-intervention measurement (n = 24). Thin grey lines represent individual participants; the thick black line represents the group mean

Comparison of H-reflex excitability between Baseline 1 and Baseline 2 revealed a significant difference (Wilcoxon signed-rank test: W = 46.0, *p* = 0.002, *r* = 0.64), indicating inherent variability in reflex measures prior to the intervention. To quantify within-session reliability of baseline H-reflex measures, an intraclass correlation coefficient (ICC; two-way mixed-effects, absolute agreement) was calculated between Baseline 1 and Baseline 2. Reliability was excellent, with ICC(3,1) = 0.93 and ICC(3,2) = 0.96 for the averaged measure. The coefficient of variation across baseline measurements was 6.75%, indicating relatively low variability. Consequently, the two baselines were averaged to form a single pre-intervention reference value (AvgBaseline) for subsequent analyses. Mean H_max_/M_max_ values were 0.612 ± 0.218 (*mean* ± *SD*) for Baseline 1, 0.657 ± 0.213 for Baseline 2, and 0.628 ± 0.220 for the Post condition. The averaged baseline (AvgBaseline) was 0.635 ± 0.208 (see Fig. 3).

**Fig. 4:**
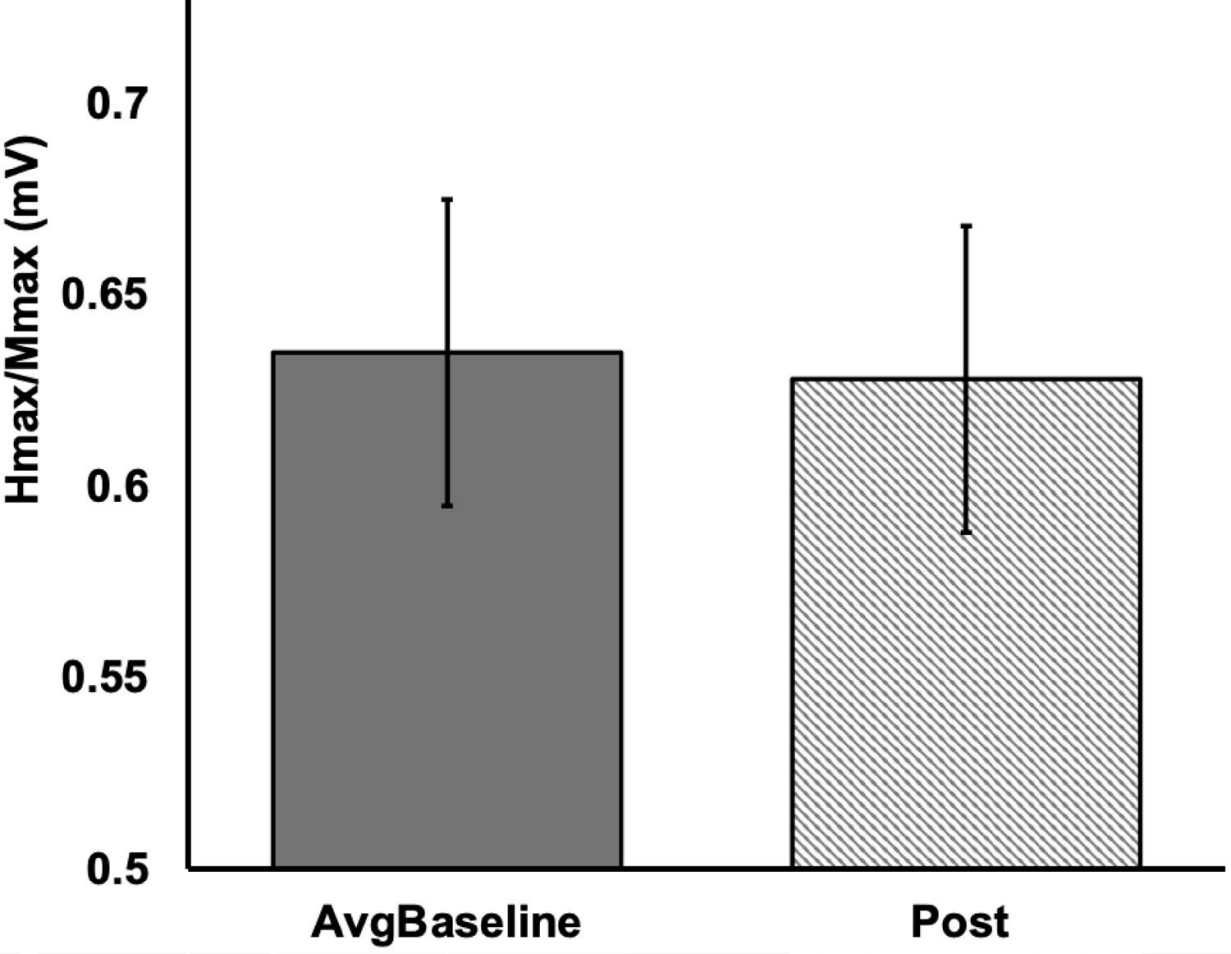
Group-level comparison of normalized soleus H-reflex excitability (H_max_/M_max_) between the averaged baseline (AvgBaseline) and post-intervention (striped) time points (n = 24). Error Bars report mean ± SE

At the group level, H_max_/M_max_ did not differ significantly between the AvgBaseline and Post measurements (W = 129.0, *p* = 0.564, *r* = 0.12; Fig. 4), suggesting that the UL movement paradigm did not elicit a consistent modulation of the soleus H-reflex across participants. This finding prompted further exploration of individual response patterns. Although variability was observed between baseline measurements, post-intervention values were comparable to the averaged baseline at the group level, while individual responses demonstrated bidirectional changes, supporting the interpretation that modulation reflects more than within-session fluctuation alone.

**Fig. 5:**
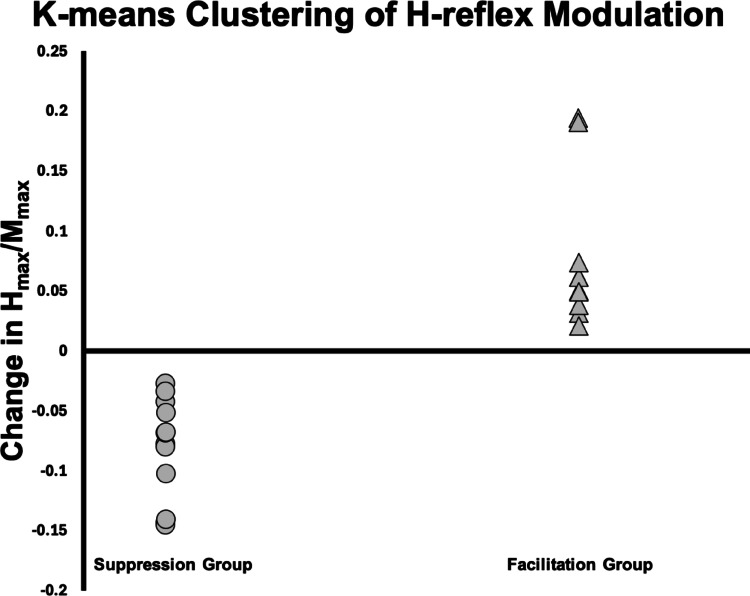
K-means clustering of individual H-reflex responses following upper limb (UL) movement. Participants were clustered based on the difference between post-intervention and average baseline H_max_/M_max_ ratios (Change in H_max_/M_max_ (mV) = Post − AvgBaseline). Two distinct response groups emerged: one exhibited suppression of spinal excitability (n = 13, shown with circles; negative values), and the other exhibited facilitation (n = 11, shown with squares; positive values)

To examine interindividual variability, k-means clustering was applied to individual change scores (Post − AvgBaseline). Based on an a priori hypothesis of bidirectional modulation (suppression vs. facilitation) and supported by both elbow and silhouette validation methods, the optimal solution was k = 2. Alternative solutions (k = 1 and k = 3) were also evaluated; however, k = 1 did not capture meaningful variability in responses, while k = 3 produced small and unstable clusters that did not represent distinct or interpretable response patterns. The resulting clusters represented two distinct response patterns: one group exhibited suppression of spinal excitability, and the other showed facilitation (Fig. 5). A Mann–Whitney U test confirmed a significant difference between these clusters (U = 143.0, *p* < 0.001, *r* = 0.83), demonstrating marked heterogeneity in reflex modulation. To determine whether baseline spinal excitability contributed to subgroup classification, a correlation analysis was conducted between average baseline H_max_/M_max_ and reflex change scores (Post – AvgBaseline). Baseline excitability was not significantly correlated with modulation magnitude (*r* = –0.14), indicating that the facilitation and suppression patterns were not driven by initial reflex size but reflected genuine task-related modulation.

Between-cluster comparisons showed significant bidirectional changes. The suppression group (*n* = 13) displayed a 22.1% ± 11.0% (*mean* ± *SD*) decrease in H-reflex excitability relative to baseline (W = 0.0, *p* < 0.001, *r* = 0.83), while the facilitation group (n = 11) demonstrated a 19.5% ± 12.5% (*mean* ± *SD*) increase (W = 0.0, *p* = 0.001, *r* = 0.78; Fig. 5). Sex distribution was comparable across subgroups: the suppression group comprised 6 females and 7 males, and the facilitation group comprised 6 females and 5 males, indicating no clear sex imbalance between groups.

**Fig. 6:**
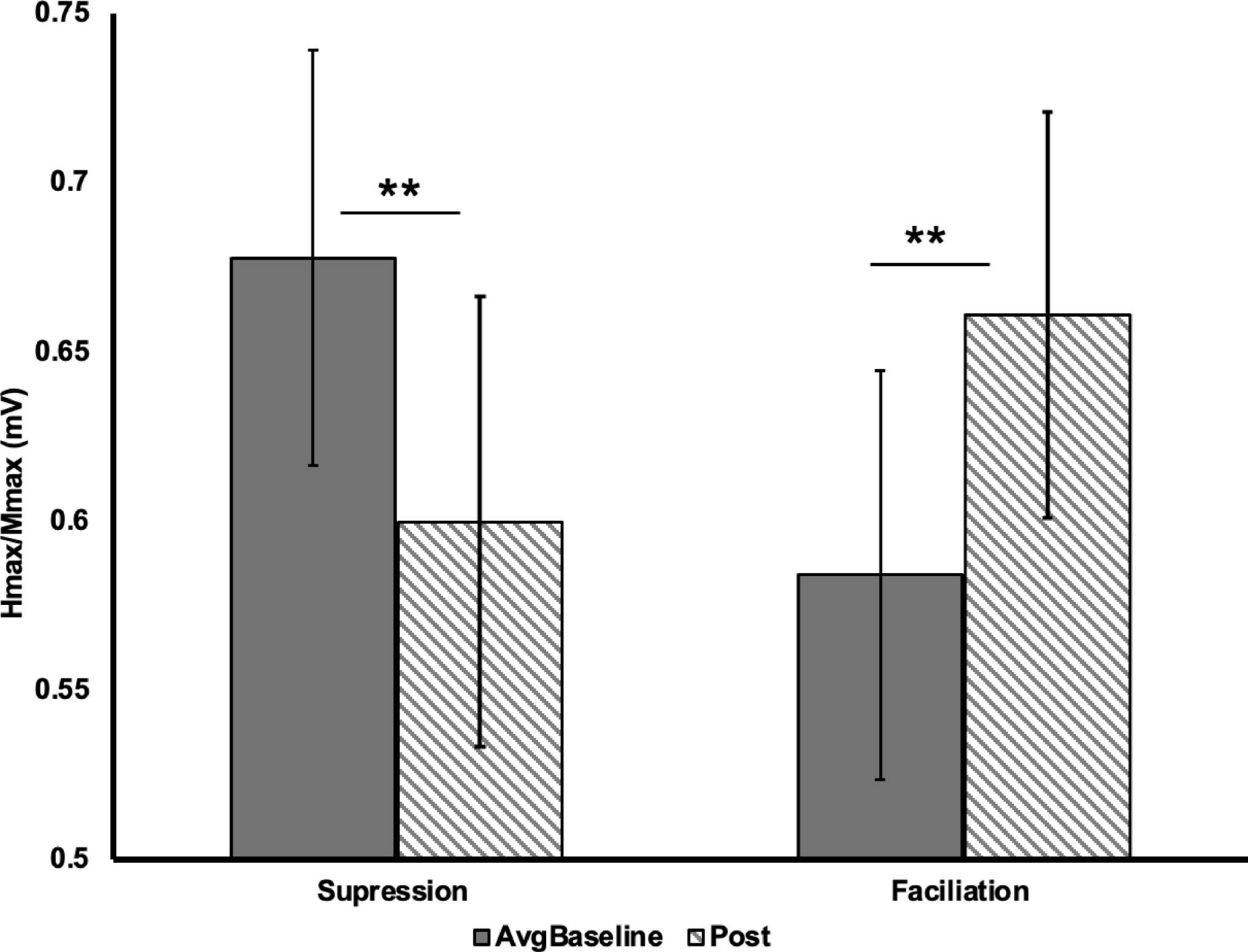
Subgroup analysis of soleus H-reflex excitability (H_max_/M_max_) between the averaged baseline (AvgBaseline) and post-intervention time points (n = 24). Bars report mean ± SE. ** *p* < 0.001

VAS-F fatigue scores increased significantly post-intervention (W = 14.0, *p* = 0.003, *r* = 0.60), whereas energy scores remained stable (W = 32.5, *p* = 0.208, *r* = 0.25), indicating that participants experienced elevated fatigue without corresponding changes in perceived energy (see Fig. 6).

## Discussion

This study investigated whether a discrete, voluntary UL movement paradigm could modulate soleus H-reflex excitability in healthy adults. No group-level change in H-reflex amplitude was observed, indicating that the intervention did not elicit a consistent direction of neuromodulatory effect across participants. However, individual-level analysis using unsupervised *k*-means clustering revealed two distinct response profiles: one subgroup showing suppression (mean 22.1% ± 11% decrease) and another showing facilitation (mean 19.5% ± 12.5% increase). These bidirectional responses demonstrate that UL-induced spinal modulation is not homogeneous but highly individualized, highlighting variability in the modulation of spinal excitability, potentially reflecting differences in descending and interlimb influences. Importantly, the magnitude of change observed in the facilitation and suppression subgroups (approximately ± 20%) was substantially greater than the within-session baseline variability observed in this study (CV = 6.75%), suggesting that the observed modulation is unlikely to be explained solely by spontaneous fluctuations. However, these subgroup classifications should be interpreted as exploratory rather than definitive neurophysiological phenotypes, and a causal relationship cannot be definitively established in the absence of a control condition.

### Individual variability and context dependence

The finding of bidirectional modulation contributes to a growing body of work emphasizing the context- and person-specific nature of interlimb neural interactions (Kato et al. 2022; Solopova et al. 2017; Zehr and Duysens 2004). Most prior research has focused on rhythmic UL paradigms, such as arm cycling, which consistently suppresses soleus H-reflex amplitude (Frigon et al. 2004; Zehr et al. 2016; Zehr and Stein 1999). By contrast, the current study extends this literature by demonstrating that even non-cyclic, symmetric UL movements can elicit either inhibitory or facilitatory changes, depending on individual neurophysiological characteristics. This heterogeneity is consistent with findings that interlimb coupling is influenced by volitional engagement, task design, and attentional demands (Javan and Zehr 2008; Solopova et al. 2017).

The significant variability between Baseline 1 and 2 observed here is consistent with previous evidence that H-reflex amplitude fluctuates within and across sessions due to spontaneous changes in motoneuron pool excitability, attention, posture, and arousal (Chen and Zhou 2011; Funase and Miles 1999; Hopkins et al. 2000; Zehr 2002). Technical factors, such as electrode micro-displacements, subtle changes in Ia afferent recruitment thresholds, and attentional factors, can further contribute to this variability (Zehr 2002).In the present study, posture, electrode placement, stimulation parameters, and testing conditions were tightly standardized to minimize extraneous influences; however, some physiological fluctuations are unavoidable. For this reason, averaging repeated baselines is widely recommended to obtain a more stable pre-intervention reference (Chen and Zhou 2011). Despite the statistically significant difference between baseline measurements, within-session reliability was high (ICC = 0.93–0.96), indicating strong consistency in relative H-reflex amplitudes across participants. The magnitude of variability (CV = 6.75%) is consistent with prior reports of soleus H-reflex reliability (ICC ≈ 0.80–0.98), particularly in standing conditions, which are typically more variable than seated paradigms (Brangaccio et al. 2025; Hopkins et al. 2000). Together, these findings suggest that the observed variability reflects expected physiological fluctuations rather than measurement error. Thus, the observed baseline differences in our facilitation and suppression subgroups likely reflect inherent physiological fluctuations rather than experimental inconsistency. Importantly, baseline H-reflex excitability did not predict whether participants exhibited suppression or facilitation, supporting the interpretation that supporting the interpretation that subgroup differences are more likely related to task-induced modulation rather than baseline-driven bias.

### Mechanistic interpretation of bidirectional responses

The bidirectional responses we observed, significant suppression in one subgroup and significant facilitation in another, indicate that UL movement can shift spinal excitability in opposite directions across individuals. Although sex differences in H-reflex excitability have been reported in previous studies (Hoffman et al. 2018; Mendonca et al. 2020), the distribution of males and females was comparable across subgroups in the present study, suggesting that sex is unlikely to account for the observed bidirectional modulation. The suppression subgroup is consistent with well-documented spinal mechanisms, such as increased presynaptic inhibition of Ia afferents and interlimb propriospinal inhibition, both of which have been classically observed during rhythmic or coordinated limb movements. Increased Ia presynaptic inhibition reduces motoneuron excitability and helps prevent excessive or conflicting reflex activation across limbs. During bilateral UL movement, synchronized sensory input from both arms may enhance this inhibition at the lumbosacral level, leading to reduced soleus H-reflex amplitude. Such interlimb inhibitory effects align with prior work showing that UL activity can down-regulate LL reflex excitability through propriospinal pathways (Capaday and Stein 1986; Frigon et al. 2007; Zehr and Stein 1999; Zehr and Duysens 2004). These circuits, which coordinate cervical and lumbosacral pattern generators to stabilize locomotor output, provide a plausible mechanistic explanation for the reflex suppression observed after UL movement practice (Zehr et al. 2016; Zehr and Duysens 2004).

Conversely, facilitation of the H-reflex may result from enhanced descending drive or reduced presynaptic inhibition. Kato et al. (2022) reported that voluntary wrist flexion increased corticospinal excitability of the soleus, whereas similar movements induced by neuromuscular electrical stimulation did not, highlighting the importance of volitional cortical engagement in interlimb circuit modulation. Similarly, Nakajima et al. (2013) and others have proposed that increased recruitment of propriospinal interneurons or heightened sensory integration can enhance the excitability of distant motor neuron pools in the spinal cord through intersegmental connectivity.

The following several overlapping mechanisms are proposed as plausible interpretations of the observed bidirectional responses; however, these remain inferential:

*Descending cortical drive.* Voluntary, attentionally guided UL movement engages motor cortical regions that increase corticospinal excitability and lower the activation threshold of both UL (targeted by the motor practice) and LL motoneurons not directly involved in the motor practice (Kato et al. 2022). Even low-intensity voluntary UL contractions can produce measurable facilitation in the soleus, underscoring the sensitivity of these pathways (Klimstra et al. 2009). For example, Klimstra et al. (2009) showed that voluntary arm movement increases corticospinal excitability to LL muscles, even at relatively low movement frequencies, supporting the idea that modest UL engagement can influence LL motoneuron responsiveness.

*Modulation of presynaptic inhibition.* Cognitive and attentional factors can modulate spinal excitability in a task-dependent manner, with evidence that changes in cognitive load, sensory context, and task demands alter soleus H-reflex amplitude in humans (e.g., Llewellyn et al. 1990; Pinar et al. 2010; Sibley et al. 2007; Weaver et al. 2012). These effects are thought to be mediated, at least in part, by modulation of Ia presynaptic inhibition via descending control mechanisms (e.g., Meunier and Pierrot-Deseilligny 1998; Perez et al. 2005). Individual differences in attentional control or arousal may therefore contribute to whether inhibition or facilitation predominates. While operant conditioning studies demonstrate that the H-reflex can be systematically increased or decreased through repeated, task-specific training (Thompson and Wolpaw 2014; Thompson et al. 2009), participants in the present study were not instructed to consciously modulate their reflex. Accordingly, the observed bidirectional changes likely reflect spontaneous, state-dependent modulation rather than learned, volitional control.

*Interlimb sensory integration.* Cutaneous and proprioceptive afferents from the UL can modulate LL motoneuron excitability at short latencies consistent with spinal transmission. Toth et al. (2022) demonstrated that hand stimulation elicited both excitatory and inhibitory effects in the soleus and tibialis anterior, suggesting that spinal interlimb coupling is capable of flexible bidirectional modulation depending on sensory context.

The discrete, rhythmically cued nature of the present UL paradigm likely accentuated attentional and cortical engagement relative to passive or continuous cycling tasks (Kato et al. 2022; Klimstra et al. 2009; Toth et al. 2022). Such intentional cortical engagement of attentional, cognitive, and motor circuits, which varies across participants, may explain inter-individual variability in suppression and facilitation, reflecting differential weighting of cortical and spinal influences. These divergent outcomes are unlikely to be random but may instead represent stable individual signatures of sensorimotor integration capacity or corticospinal reserve. Collectively, these findings support a conceptual framework in which discrete upper limb movements bias spinal excitability through interacting cortical, spinal, and sensory pathways, with the net effect (facilitation or suppression) determined by the relative weighting of these mechanisms across individuals. Future work can investigate individual characteristics that influence the magnitude and direction of sensorimotor integration, as well as examine these circuits in individuals with neurological deficits.

### Clinical and research implications

As this study was conducted in healthy young adults within a single experimental session, direct clinical translation remains to be established. Accordingly, caution is warranted when interpreting clinical relevance. The clustering analysis in this study was exploratory and intended to identify patterns of response rather than to define stable neurophysiological phenotypes or directly link individuals to specific functional mechanisms. The coexistence of facilitation and suppression challenges the traditional assumption that UL movement uniformly inhibits LL reflexes. Our findings may have implications for rehabilitation strategies that utilize arm movements to modulate spinal excitability and LL motor control in individuals with neurological injuries. Interventions such as arm cycling are often designed to exploit reflex inhibition to reduce spasticity or improve coordination in people with spinal cord injury or stroke (Barzi and Zehr 2008; Klarner et al. 2016; Mezzarane et al. 2014). However, accumulating evidence, including the present findings, indicates that responses to UL-driven modulation are not consistent across individuals.

Rather than applying a uniform paradigm, rehabilitation approaches may benefit from further investigation into personalized strategies that tailor intervention parameters to an individual’s neural excitability profile. Zehr et al. (2016) emphasized the translational potential of exploiting interlimb coupling for rehabilitation and highlighted inter-individual variability in reflex modulation. Consistent with this, primary studies demonstrate that reflex excitability is modulated in a task- and parameter-dependent manner, with variability across individuals, including bidirectional reflex responses under similar conditions (Hundza and Zehr 2009; Mezzarane et al. 2014; Pinar et al. 2010; Sibley et al. 2007). Building on this, the current study’s classification of facilitatory and suppressive responders provides a preliminary framework for exploring adapting rehabilitation strategies based on individual neural responses.

Notably, our results showed that VAS-F fatigue scores increased following the UL movement task, while energy levels remained unchanged. This dissociation between fatigue and energy self-reports suggests that while the task may have been physically or cognitively fatiguing, it did not diminish the individual’s motivational state. Fatigue has been shown to alter spinal excitability and central drive, with evidence from human studies indicating that fatiguing contractions can increase presynaptic inhibition and depress H-reflex amplitude in a task- and time-dependent manner (Baudry et al. 2011; Kolosova and Slivko 2006; Nordlund et al. 2004). Although fatigue was measured post-intervention and not concurrently with reflex testing, its interaction with neural excitability warrants further investigation.

### Limitations and future directions

The moderate sample size (n = 24) limits generalization, and mechanistic interpretations remain inferential without direct neurophysiological assays. Additionally, clustering was performed on a single outcome variable (Post − Baseline), which may bias the separation into positive and negative responders and increase the risk of overinterpretation. Future studies should incorporate larger samples and additional techniques, such as transcranial magnetic stimulation, paired reflex depression, or somatosensory evoked potentials, to disentangle corticospinal from presynaptic contributions. Although the study did not include a no-movement control or sham condition, limiting the ability to definitively attribute changes in H-reflex excitability to the UL movement task, the use of two baseline measurements partially mitigates this limitation by accounting for time-dependent fluctuations. However, spontaneous drift in spinal excitability cannot be fully excluded. Additionally, H-reflex measurements were obtained within a single session; therefore, between-day reliability of individual modulation patterns was not assessed. Future studies should examine the reproducibility of these responses across multiple sessions to better support their use in individualized rehabilitation approaches. Finally, longitudinal designs will be essential to determine whether these effects represent transient state changes or durable plasticity.

## Conclusions

In summary, our study showed that a 20-min task practice comprising discrete, voluntary, clinically-relevant UL movements can suppress or facilitate soleus H-reflex excitability, revealing individual-specific patterns of interlimb spinal plasticity. These results may challenge the assumptions of uniform reflex suppression during or following interlimb activity, and highlight the need for personalized approaches to neuromodulation and rehabilitation grounded in information about each individual’s neural circuit excitability and responsiveness.

## Data Availability

No datasets were generated or analysed during the current study.

## References

[CR1] Arya KN, Pandian S (2014) Interlimb neural coupling: implications for poststroke hemiparesis. Ann Phys Rehabil Med 57(9–10):696–713. 10.1016/j.rehab.2014.06.003 25262645 10.1016/j.rehab.2014.06.003

[CR2] Barzi Y, Zehr EP (2008) Rhythmic arm cycling suppresses hyperactive soleus H-reflex amplitude after stroke. Clin Neurophysiol 119(6):1443–1452. 10.1016/J.CLINPH.2008.02.016 18411072 10.1016/j.clinph.2008.02.016

[CR3] Baudry S, Maerz AH, Gould JR, Enoka RM (2011) Task- and time-dependent modulation of Ia presynaptic inhibition during fatiguing contractions performed by humans. J Neurophysiol 106(1):265. 10.1152/JN.00954.2010 21543747 10.1152/jn.00954.2010 PMC3129736

[CR4] Brangaccio JA, Gupta D, Mojtabavi H, Hardesty RL, Hill NJ, Carp JS, Wolpaw JR (2025) Soleus H-reflex size versus stimulation rate in the presence of background muscle activity: a methodological study. *BioRxiv*, 2025.03.17.643784. 10.1101/2025.03.17.643784 10.1007/s00221-025-07149-x PMC12679875 40974412

[CR5] Burke D (2016) Clinical uses of H reflexes of upper and lower limb muscles. Clin Neurophysiol Pract 1:9–17. 10.1016/J.CNP.2016.02.003 30214954 10.1016/j.cnp.2016.02.003 PMC6123946

[CR6] Burke RE (1999) The use of state-dependent modulation of spinal reflexes as a tool to investigate the organization of spinal interneurons. Exp Brain Res 128(3):263–277. 10.1007/S002210050847/METRICS 10501799 10.1007/s002210050847

[CR7] Capaday C, Stein RB (1986) Amplitude modulation of the soleus H-reflex in the human during walking and standing. J Neurosci 6(5):1308–1313. 10.1523/JNEUROSCI.06-05-01308.1986 3711981 10.1523/JNEUROSCI.06-05-01308.1986 PMC6568550

[CR8] Chen YS, Zhou S (2011) Soleus H-reflex and its relation to static postural control. Gait Posture 33(2):169–178. 10.1016/J.GAITPOST.2010.12.008 21211976 10.1016/j.gaitpost.2010.12.008

[CR9] Collins SH, Adamczyk PG, Kuo AD (2009) Dynamic arm swinging in human walking. Proc Biol Sci 276(1673):3679–3688. 10.1098/RSPB.2009.0664 19640879 10.1098/rspb.2009.0664 PMC2817299

[CR10] Delwaide PJ, Figiel C, Richelle C (1977) Effects of postural changes of the upper limb on reflex transmission in the lower limb. Cervicolumbar reflex interactions in man. J Neurol Neurosurg Psychiatry 40(6):616–621. 10.1136/JNNP.40.6.616 903777 10.1136/jnnp.40.6.616 PMC492771

[CR11] Frigon A, Carroll TJ, Jones KE, Zehr EP, Collins DF (2007) Ankle position and voluntary contraction alter maximal M waves in soleus and tibialis anterior. Muscle Nerve 35(6):756–766. 10.1002/MUS.20747 17295303 10.1002/mus.20747 PMC5005069

[CR12] Frigon A, Collins DF, Zehr EP (2004) Effect of rhythmic arm movement on reflexes in the legs: modulation of soleus H-reflexes and somatosensory conditioning. J Neurophysiol 91(4):1516–1523. 10.1152/jn.00695.2003 14657191 10.1152/jn.00695.2003

[CR13] Funase K, Miles TS (1999) Observations on the variability of the H reflex in human soleus. Muscle Nerve 22(3):341–346. 10.1002/(SICI)1097-4598(199903)22:3<341::AID-MUS6>3.0.CO;2-R 10086894 10.1002/(sici)1097-4598(199903)22:3<341::aid-mus6>3.0.co;2-r

[CR14] Hoffman M, Norcross M, Johnson S (2018) The Hoffmann reflex is different in men and women. NeuroReport 29(4):314–316. 10.1097/WNR.0000000000000961 29293170 10.1097/WNR.0000000000000961

[CR15] Hopkins JT, Ingersoll CD, Cordova ML, Edwards JE (2000) Intrasession and intersession reliability of the soleus H-reflex in supine and standing positions. Electromyogr Clin Neurophysiol 40(2):89–94 10746184

[CR16] Hoque M, Ardizzone M, Sabatier M, Borich M, Kesar T (2018) Longer duration of downslope treadmill walking induces depression of H-reflexes measured during standing and walking. Neurology PMC6483108 31032493

[CR17] Hoque M, Borich M, Sabatier M, Backus D, Kesar T (2019) Effects of downslope walking on Soleus H-reflexes and walking function in individuals with multiple sclerosis: a preliminary study. NeuroRehabilitation 44(4):587–597. 10.3233/NRE-192701 31256089 10.3233/NRE-192701

[CR18] Hundza SR, Zehr EP (2009) Suppression of soleus H-reflex amplitude is graded with frequency of rhythmic arm cycling. Exp Brain Res 193(2):297–306. 10.1007/S00221-008-1625-0 19011847 10.1007/s00221-008-1625-0

[CR19] Javan B, Zehr EP (2008) Short-term plasticity of spinal reflex excitability induced by rhythmic arm movement. J Neurophysiol 99(4):2000–2005. 10.1152/JN.01315.2007 18234977 10.1152/jn.01315.2007

[CR20] Kato T, Kaneko N, Sasaki A, Endo N, Yuasa A, Milosevic M, Nakazawa K (2022) Corticospinal excitability and somatosensory information processing of the lower limb muscle during upper limb voluntary or electrically induced muscle contractions. Eur J Neurosci 55(7):1810–1824. 10.1111/ejn.15643 35274383 10.1111/ejn.15643

[CR21] Klarner T, Barss TS, Sun Y, Kaupp C, Loadman PM, Zehr EP (2016) Exploiting interlimb arm and leg connections for walking rehabilitation: a training intervention in stroke. Neural Plast. 10.1155/2016/1517968 27403344 10.1155/2016/1517968 PMC4926010

[CR22] Klimstra MD, Thomas E, Stoloff RH, Ferris DP, Zehr EP (2009) Neuromechanical considerations for incorporating rhythmic arm movement in the rehabilitation of walking. Chaos 19(2):26102. 10.1063/1.3147404 10.1063/1.3147404 19566262

[CR23] Knikou M (2007) Neural coupling between the upper and lower limbs in humans. Neurosci Lett 416(2):138–143. 10.1016/j.neulet.2007.01.072 17331647 10.1016/j.neulet.2007.01.072

[CR24] Knikou M (2008) The H-reflex as a probe: pathways and pitfalls. J Neurosci Methods 171(1):1–12. 10.1016/J.JNEUMETH.2008.02.012 18394711 10.1016/j.jneumeth.2008.02.012

[CR25] Kolosova EV, Slivko ÉI (2006) Fatigue-induced modulation of the H reflex of soleus muscle in humans. Neurophysiology 38(5):360–364. 10.1007/S11062-006-0072-4

[CR26] Lee KA, Hicks G, Nino-Murcia G (1991) Validity and reliability of a scale to assess fatigue. Psychiatry Res 36(3):291–298. 10.1016/0165-1781(91)90027-M 2062970 10.1016/0165-1781(91)90027-m

[CR27] Llewellyn M, Yang JF, Prochazka A (1990) Human H-reflexes are smaller in difficult beam walking than in normal treadmill walking. Exp Brain Res 83(1):22–28. 10.1007/BF00232189 2073943 10.1007/BF00232189

[CR28] Mendonca GV, Pezarat-Correia P, Gonçalves AD, Gomes M, Correia JM, Vila-Chã C (2020) Sex differences in soleus muscle H-reflex and V-wave excitability. Exp Physiol 105(11):1928–1938. 10.1113/EP088820 32886814 10.1113/EP088820

[CR29] Meunier S, Pierrot-Deseilligny E (1998) Cortical control of presynaptic inhibition of Ia afferents in humans. Exp Brain Res 119(4):415–426. 10.1007/S002210050357 9588776 10.1007/s002210050357

[CR30] Mezzarane RA, Nakajima T, Zehr EP (2014) After stroke bidirectional modulation of soleus stretch reflex amplitude emerges during rhythmic arm cycling. Front Hum Neurosci 8(MAR):136. 10.3389/FNHUM.2014.00136/BIBTEX 24701201 10.3389/fnhum.2014.00136 PMC3965852

[CR31] Nakajima T, Mezzarane RA, Klarner T, Barss TS, Hundza SR, Komiyama T, Zehr EP (2013) Neural mechanisms influencing interlimb coordination during locomotion in humans: presynaptic modulation of forearm H-reflexes during leg cycling. PLoS ONE 8(10):e76313. 10.1371/journal.pone.0076313 24204611 10.1371/journal.pone.0076313 PMC3799938

[CR32] Nordlund MM, Thorstensson A, Cresswell AG (2004) Central and peripheral contributions to fatigue in relation to level of activation during repeated maximal voluntary isometric plantar flexions. J Appl Physiol 1985 96(1):218–225. 10.1152/JAPPLPHYSIOL.00650.2003 12972439 10.1152/japplphysiol.00650.2003

[CR33] Palmieri RM, Ingersoll CD, Hoffman MA (2004) The Hoffmann Reflex: methodologic considerations and applications for use in sports medicine and athletic training research. J Athl Train 39(3):268 16558683 PMC522151

[CR34] Palomino AF, Hundza SR, Zehr EP (2011) Rhythmic arm cycling differentially modulates stretch and H-reflex amplitudes in soleus muscle. Exp Brain Res 214(4):529–537. 10.1007/S00221-011-2851-4 21901451 10.1007/s00221-011-2851-4

[CR35] Perez MA, Lungholt BKS, Nielsen JB (2005) Presynaptic control of group Ia afferents in relation to acquisition of a visuo-motor skill in healthy humans. J Physiol 568(Pt 1):343–354. 10.1113/JPHYSIOL.2005.089904 16051628 10.1113/jphysiol.2005.089904 PMC1474778

[CR36] Pinar S, Kitano K, Koceja DM (2010) Role of vision and task complexity on soleus H-reflex gain. J Electromyogr Kinesiol 20(2):354–358. 10.1016/J.JELEKIN.2009.03.002 19356950 10.1016/j.jelekin.2009.03.002

[CR37] Sibley KM, Carpenter MG, Perry JC, Frank JS (2007) Effects of postural anxiety on the soleus H-reflex. Hum Mov Sci 26(1):103–112. 10.1016/j.humov.2006.09.004 17137663 10.1016/j.humov.2006.09.004

[CR38] Solopova IA, Selionov VA, Blinov EO, Zhvansky DS, Ivanenko YP (2017) Rhythmic wrist movements facilitate the soleus H-reflex and non-voluntary air-stepping in humans. Neurosci Lett 638:39–45. 10.1016/j.neulet.2016.12.007 27931775 10.1016/j.neulet.2016.12.007

[CR39] Thompson AK, Wolpaw JR (2014) Operant conditioning of spinal reflexes: from basic science to clinical therapy. Front Integr Neurosci 8(MAR):80098. 10.3389/FNINT.2014.00025/BIBTEX 10.3389/fnint.2014.00025 PMC3957063 24672441

[CR40] Thompson AK, Xiang YC, Wolpaw JR (2009) Acquisition of a simple motor skill: task-dependent adaptation plus long-term change in the human soleus H-reflex. J Neurosci off J Soc Neurosci 29(18):5784–5792. 10.1523/JNEUROSCI.4326-08.2009 10.1523/JNEUROSCI.4326-08.2009 PMC2696311 19420246

[CR41] Toth AL, Fenrich KK, Jones KE, Misiaszek JE (2022) Coupling of single cutaneous afferents in the hand with ankle muscles, and their response to rapid light touch displacements. J Neurophysiol 127(4):1040–1053. 10.1152/jn.00280.2021 35320053 10.1152/jn.00280.2021

[CR42] Weaver TB, Janzen MR, Adkin AL, Tokuno CD (2012) Changes in spinal excitability during dual task performance. J Mot Behav 44(4):289–294. 10.1080/00222895.2012.702142 22856330 10.1080/00222895.2012.702142

[CR43] Zehr EP, Barss TS, Dragert K, Frigon A, Vasudevan EV, Haridas C, Hundza S, Kaupp C, Klarner T, Klimstra M, Komiyama T, Loadman PM, Mezzarane RA, Nakajima T, Pearcey GEP, Sun Y (2016) Neuromechanical interactions between the limbs during human locomotion: An evolutionary perspective with translation to rehabilitation. Exp Brain Res 234(11):3059–3081. 10.1007/s00221-016-4715-4 27421291 10.1007/s00221-016-4715-4 PMC5071371

[CR44] Zehr EP (2002) Considerations for use of the Hoffmann reflex in exercise studies. Eur J Appl Physiol 86(6):455–468. 10.1007/S00421-002-0577-5/METRICS 11944092 10.1007/s00421-002-0577-5

[CR45] Zehr EP (2005) Neural control of rhythmic human movement: the common core hypothesis. Exerc Sport Sci Rev 33(1):54–60 15640722

[CR46] Zehr EP, Carroll TJ, Chua R, Collins DF, Frigon A, Haridas C, Thompson AK (2004) Possible contributions of CPG activity to the control of rhythmic human arm movement, 82(8–9), 556–568 10.1139/y04-056 15523513

[CR47] Zehr EP, Haridas C (2003) Modulation of cutaneous reflexes in arm muscles during walking: Further evidence of similar control mechanisms for rhythmic human arm and leg movements. Exp Brain Res 149(2):260–266. 10.1007/S00221-003-1377-9 12610695 10.1007/s00221-003-1377-9

[CR48] Zehr EP, Hundza SR, Vasudevan EV (2009) The quadrupedal nature of human bipedal locomotion. Exerc Sport Sci Rev 37(2):102–108. 10.1097/JES.0B013E31819C2ED6 19305202 10.1097/JES.0b013e31819c2ed6

[CR49] Zehr EP, Stein RB (1999) What functions do reflexes serve during human locomotion? Prog Neurobiol 58(2):185–205. 10.1016/S0301-0082(98)00081-1 10338359 10.1016/s0301-0082(98)00081-1

[CR50] Zehr EP, Duysens J (2004) Regulation of arm and leg movement during human locomotion. Neuroscientist 10(4):347–361. 10.1177/1073858404264680 15271262 10.1177/1073858404264680

